# Selective Blockade of Trypanosomatid Protein Synthesis by a Recombinant Antibody Anti-*Trypanosoma cruzi* P2β Protein

**DOI:** 10.1371/journal.pone.0036233

**Published:** 2012-05-03

**Authors:** Maximiliano Juri Ayub, Benson Nyambega, Leandro Simonetti, Tomas Duffy, Silvia A. Longhi, Karina A. Gómez, Johan Hoebeke, Mariano J. Levin, Cristian R. Smulski

**Affiliations:** 1 Laboratorio de Biología Molecular de la Enfermedad de Chagas, Instituto de Investigaciones en Ingeniería Genética y Biología Molecular, Consejo Nacional de Investigaciones Científicas y Técnicas, Buenos Aires, Argentina; 2 Instituto Multidisciplinario de Investigaciones Biológicas de San Luis, Consejo Nacional de Investigaciones Científicas y Técnicas, Universidad Nacional de San Luis, San Luis, Argentina; 3 Department of Medical Biochemistry, Maseno University, Kisumu, Kenya; 4 Zentrum für Molekulare Biologie der Universität Heidelberg, Heidelberg, Germany; 5 Immunologie et Chimie Thérapeutiques (UPR 9021), Institut de Biologie Moleculaire et Cellulaire (IBMC), Strasbourg, France; Louisiana State University, United States of America

## Abstract

The ribosomal P proteins are located on the stalk of the ribosomal large subunit and play a critical role during the elongation step of protein synthesis. The single chain recombinant antibody C5 (scFv C5) directed against the C-terminal region of the *Trypanosoma cruzi* P2β protein (TcP2β) recognizes the conserved C-terminal end of all *T. cruzi* ribosomal P proteins. Although this region is highly conserved among different species, surface plasmon resonance analysis showed that the scFv C5 possesses very low affinity for the corresponding mammalian epitope, despite having only one single amino-acid change. Crystallographic analysis, *in silico* modelization and NMR assays support the analysis, increasing our understanding on the structural basis of epitope specificity. *In vitro* protein synthesis experiments showed that scFv C5 was able to specifically block translation by *T. cruzi* and *Crithidia fasciculata* ribosomes, but virtually had no effect on *Rattus norvegicus* ribosomes. Therefore, we used the scFv C5 coding sequence to make inducible intrabodies in *Trypanosoma brucei*. Transgenic parasites showed a strong decrease in their growth rate after induction. These results strengthen the importance of the P protein C terminal regions for ribosomal translation activity and suggest that trypanosomatid ribosomal P proteins could be a possible target for selective therapeutic agents that could be derived from structural analysis of the scFv C5 antibody paratope.

## Introduction

*Trypanosoma cruzi* is a protozoan parasite responsible for Chagas' disease. This is an endemic disease in Latin America that affects 18–20 million people. No vaccines are available at present and drugs used for treatment show undesirable side effects. The identification of new targets for chemotherapy is a major challenge in the control of the disease and the protein synthesis machinery has been proven to be such a target in other species. Insight into the mechanism capable of selectively blocking protein synthesis could thus lead to the discovery of new therapeutic agents.

**Figure 1:**
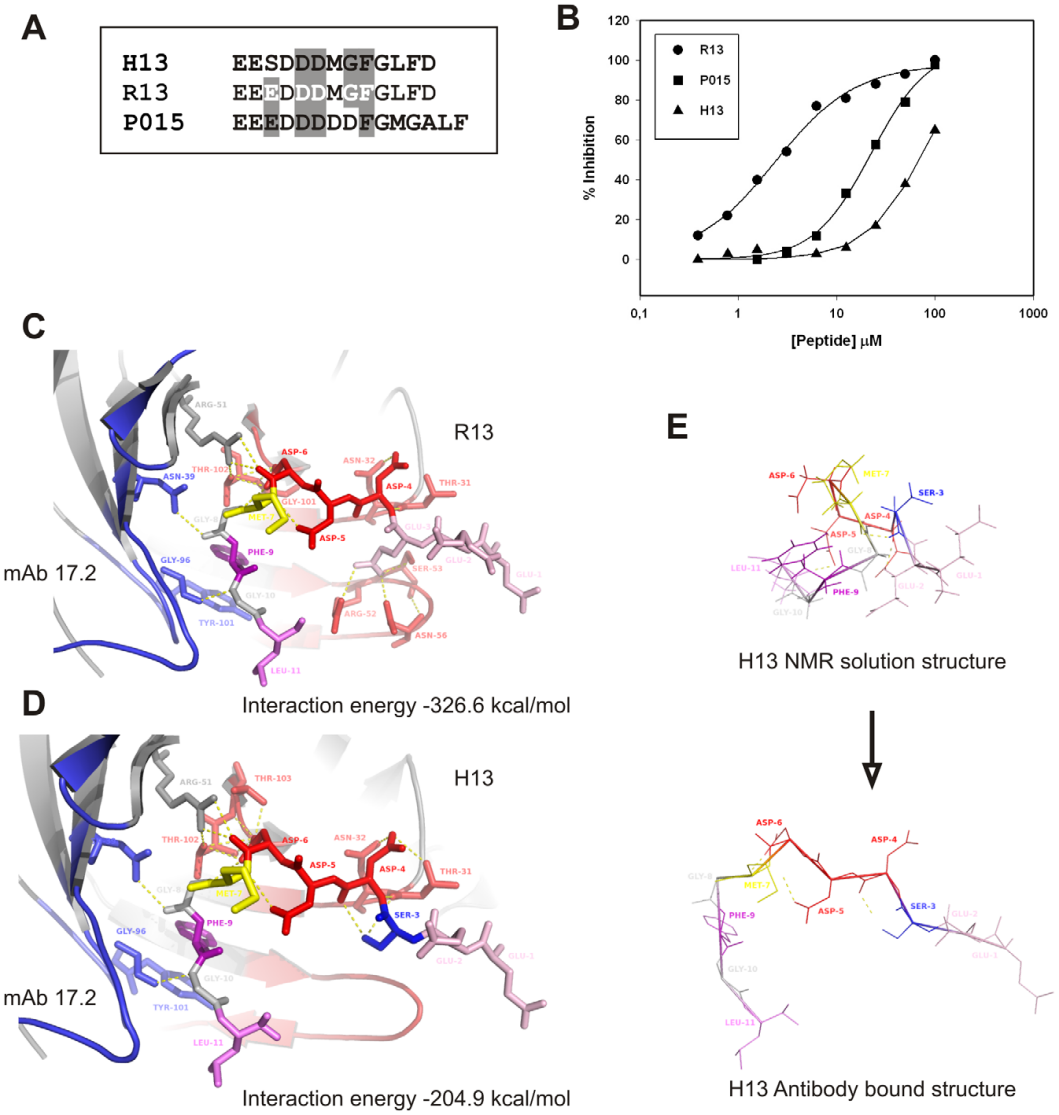
scFv C5 Epitope specificity. **A.** Sequence aligment of *T. cruzi* R13 and P015 peptides with the mammalian counterpart H13 peptide. White letters correspond to residues necessary for antibody recognition, as identified by alanine scanning. Grey background corresponds to those residues conserved in the other two peptides. **B.** Inhibition of the interaction between scFv C5 and TcP2β protein by R13, P015 and H13 peptides, using surface plasmon analysis. The figure corresponds to one representative result out of 3 independent assays. **C.** Crystal structure of the complex mAb 17.2-R13 (PDB 3SGE). **D.** Model of the interaction of mAb 17.2 with the H13 peptide. The interaction energy is indicated on each case. **E.** NMR solution structure of H13 peptide (PDB 1S4J) in comparison with the antibody-bound peptide structure modeled.

The large subunit of the eukaryotic ribosome possesses a long and protruding stalk formed by the ribosomal P proteins. These proteins include P0, an approximately 34 kDa polypeptide, and two distinct, but closely related peptides of about 11 kDa, P1 and P2. All of them share a conserved, highly acidic motif at its C-terminal end. An additional P protein, named P3, has been described in plants [1]. The number of P1/P2 subtypes varies among species. In higher eukaryotes, the P1 and P2 families have only one member. In *Saccharomyces cerevisiae*, these families have two members, P1α/P1β and P2α/P2β [2]. *Trypanosoma cruzi* also possesses two different P1 and P2 proteins [3], [4]. Interestingly, the *T. cruzi* P0 protein has a C-terminal end that deviates from the eukaryotic P consensus and bears similarity to that of the L10 protein of Archaea [5]. The GTPase activity of the eukaryotic elongation factor 2 (eEF-2), which catalyses the translocation of peptidyl-tRNA from the A to the P site of the ribosome, is dependent on the presence of P proteins on the large ribosomal subunit [6]. Specifically, the C-terminal region of the ribosomal P proteins was shown to be essential during this step [7], [8]. Thus, the ribosomal stalk is directly involved in the translocation step of protein synthesis [9]. It has been previously shown that antibodies against the C-terminal region of ribosomal P proteins (markers of systemic lupus erythematosus in humans) and their scFv recombinant forms posses the ability to block *in vitro* translation in a rabbit reticulocyte lysate system [10], [11]. In chronic Chagas' heart disease, antibodies against the C-terminal region of *T. cruzi* ribosomal P proteins have been also detected [12], [13]. However, fine epitope mapping demonstrated that the specificity of the antibodies induced in these two pathological disorders is different [14], [15]. The single chain recombinant antibody (scFv) C5 directed against the C-terminal region of the ribosomal P2β protein of *T. cruzi* (R13 epitope), targets the five P proteins that constitute the stalk [16], [17]. Four of them (P1α, P1β, P2α, P2β) contain the same C-terminal epitope, R13 (Figure 1A); and the fifth, P0, has a closely related epitope called P015 (Figure 1A) [3], [16], [18]. This antibody however, as shown in this work, possesses very low affinity for the corresponding mammalian epitope (H13) that has one single non-conservative amino acid change in the third residue. We found that the scFv C5 was able to specifically block *in vitro* protein synthesis by trypanosomatid ribosomes, but had virtually no effect on translation by mammalian ribosomes. We expressed for the first time an intrabody (intracellular antibody), derived from scFv C5, in trypanosomatid cells resulting in growth arrest. Therefore, we propose the ribosomal stalk as a novel potential chemotherapeutic target, and the scFv C5 paratope as a model for peptide mimetics synthesis for selective blocking of the parasite protein synthesis apparatus.

## Materials and Methods

### Synthetic peptides

Peptides were prepared by the solid-phase method of Merrifield as was previously described [19], using a semiautomatic multisynthesizer NPS 4000 (NeoMPS SA, Strasbourg, France).

### Surface Plasmon Resonance

The BIACORE 3000 system, sensor chip CM5, surfactant P20, amine coupling kit containing N-hydroxysuccinimide (NHS) and N-Ethyl-N′-dimethylaminopropyl carbodiimide (EDC), ethanolamine were from BIACORE (Uppsala, Sweden). Biosensor assays were performed with HBS-EP buffer as running buffer (10 mM HEPES, 150 mM sodium chloride, 3 mM EDTA, 0.005% (v/v) surfactant P20, pH 7.4). The low carboxylated dextran matrix (B1) was activated with 35 µl of a mixture 0.2 M N-ethyl-N-dimethylaminopropyl carbodiimide and 0.05 M N-hydroxysuccinimide at 5 µl/min. TcP2β-GST fusion protein and GST (as control) were immobilized with the standard BIACORE protocol at a density of 0.05 pmol/mm2 [4]. The scFv C5 was then pre-incubated with different concentrations of P015, R13 or H13 peptides for 30 min and then injected on the sensor chip for 2 min, followed by a dissociation phase of 3 min at a flow rate of 30 µl/min. The sensor chip surface was regenerated after each experiment by injecting 20 µL of 3 M MgCl_2_. From the decrease in the initial linear kinetics of the interaction in the presence of increasing amounts of inhibitor, the IC_50_ could be determined for each inhibitor as previously described [16].

### ScFv C5 DNA constructs and intrabody expression

Expression of scFv C5 in *E. coli* and purification was performed as previously described [16]. For *in vivo* expression, the DNA encoding for scFv C5 was amplified by PCR using the following oligonucleotides carrying HindIII restriction site (C5ForHIND: 5′-GT AAGCTT GCC ATG GCC GAA GTG CAG C-3′) and BamHI restriction site (C5RevBAM: 5′-GT GGATCC CCG TTT TAT TTC CAG CTT GGT CC-3′) and inserted into pHD1700 vector as previously described [20]. The N-terminal myc-tagged scFv C5 construct was transfected into blood stream 1313-514 *T. brucei* inducible cell line [21], expressed and detected by Western blot using peroxidase anti-myc monoclonal antibody (1∶50,000) (Santa cruz biotechnology). Monoclonal antibody to aldolase [22] (1∶4000) was used to detect aldolase, used as the loading control. For growth rate analysis, parasites were seeded at 2×10^5^ cells/ml and counted every 24 hours. The concentration was adjusted (by diluting appropriately) every 24 hours back to 2×10^5^ cells/ml. The cumulative number of parasites corresponds to the number of parasites per milliliter multiplied by the dilution factor for each day (day after day).

### Molecular modeling

All procedures were performed with Discovery Studio 2.5 software from Accelrys (San Diego, CA, USA). The scFv C5 model derives from the parental mAb 17.2 crystal structure Apo (3SGD) or in complex with R13 peptide (3SGE). The former structure was used as template to build a model antibody-H13 interaction. For this purpose, we mutated the aspartic acid 3 of R13 by serine and optimized the conformation of both the mutated residues and any surrounding residues that lay within a cut-off radius of 2 Å. Five models thus obtained were scored by the Discrete Optimized Protein Energy (DOPE). We continued analysis using the lowest energy model (DOPE = −96583.96 kcal/mol). Finally, the complex was subjected to a minimization step (max 500), RMS gradient 0.1 Kcal/mol by conjugated gradient.

### Gel electrophoresis and *Western blot* analysis

SDS-PAGE analysis was performed as a standard procedure using 12% acrylamide gels followed by immunoblotting. Proteins were transferred from gels onto a Hybond ECL nitrocellulose transfer membrane (Amersham Pharmacia, UK) using a mini trans-blot system (Bio-Rad, Hercules, CA, USA) in transfer buffer (25 mM Tris-HCl, 190 mM glycine, 20% (v/v) methanol, pH 8.3). Membranes were soaked in PBS-T (20 mM Na_2_HPO_4_, 1.8 mM K_2_HPO_4_, 150 mM NaCl, 2.7 mM KCl, 0.1% Tween 20, pH 7.4) supplemented with 5% (p/v) non-fat milk powder. This was followed by incubation with scFv C5 200 nM together with peroxidase-conjugated anti-His Ab (∼350 nM) (Sigma, St. Louis, MO, USA), for detection of ribosomal P proteins and with peroxidase-conjugated anti-His Ab (∼350 nM) alone for intrabody detection. The Ab was diluted in the blocking solution PBS-T 1% (p/v) non-fat milk powder. Proteins on transferred membranes were revealed with tetramethyl-benzidine (TMB) (Sigma, St. Louis, MO, USA) after a brief wash with dextran sulphate 1% (Sigma, St. Louis, MO, USA).

### Ribosome purification

Purification of ribosomes from different sources and *in vitro* protein synthesis assays were performed as described [23]. Briefly, mammalian ribosomes were obtained from rat liver (20 g), which was washed in sucrose 0.25 M and then homogenized in buffer containing 50 mM Tris–HCl, pH 7.5; 250 mM KCl; 5 mM magnesium acetate; sucrose 0.25 M and supplemented with 10% of S_150_ fraction [23]. The homogenate was treated with 10 U/ml of a-amylase and 0.1 mM CaCl_2_ for 15 min at 4°C, and then centrifuged for 4 min at low speed. The supernatant was again centrifuged for 20 min at 23,000 g. The pellet was discarded and the supernatant containing polysomes was supplemented with Triton X-100 1%, deoxycholate 0.5% and centrifuged for 5 min at 16,000 g. The new supernatant fluid (around 12 ml) was layered onto two layers of 2 M and 1.5 M sucrose made up in Buffer A (50 mM Tris–HCl, pH 7.5; 5 mM magnesium acetate; 250 mM KCl, dithiothreitol 1 mM and 10% of S_150_ fraction) and centrifuged 16 h at 140,000 g. The pellet corresponding to polysomes was rinsed and resuspended in a buffer containing 10 mM Tris–HCl, pH 7.5; 10 mM KCl and 1.5 mM magnesium acetate. Ribosome concentration was determined by optical density at 260 nm.

Polysomes from trypanosomatids were obtained from cultures at the exponential phase of growing. Cultures were treated with cycloheximide (50 ug/ml) for 10 min before harvesting by centrifugation at 4°C. Cells were washed twice with PBS containing 50 ug/ml cycloheximide. The cells were resuspended in Lysis Buffer (20 mM Tris–HCl, pH 7.5; 1 mM MgCl_2_; 5 mM KCl; 3 mM CaCl_2_; 5 mM 2-merchaptoethanol and 250 mM sucrose) and lysed with 0.2–0.4% of Nonidet P40 at 4°C. The homogenate was centrifuged 2–3 times for 20 min at 1,000 g and the final supernatant fraction containing ribosomes was layered onto a discontinuous gradient of 2 and 1.5 M of sucrose made up in the following buffer: 10 mM Tris–HCl, pH 7.5; 1 mM magnesium acetate; and 100 mM potasium acetate. The gradient was centrifuged for 16 h at 140,000 g. The supernatant was discarded and the pellet carefully rinsed and resuspended as described above.

### *In vitro* protein synthesis assays

The reaction mixtures were prepared on ice and contained: 19 amino acids 50 uM each (excepting Met); 2 mM dithiothreitol; 100 mM potassium acetate; 3.5 mM magnesium acetate; 75 ug/ml wheat germ tRNA; 18 mM Hepes/KOH, pH 7.5; 1 mM ATP; 0.5 mM GTP; 7.5 mM creatine phosphate; 37.5 ug/ml creatine phosphokinase; rat liver S_150_ fraction (24 ug of protein); 0.3 A260U of ribosomes and 2 uCi of [^35^S] methionine in a final volume of 30 ul. Reactions were performed at 30°C during 60 min and stopped by adding 150 ul of 1.5 M NaOH; 1 mM Met, 170 ug/ml BSA. After incubation for 30 min at 37°C, proteins were precipitated with 1 ml of cold TCA 25%. After 60 min on ice, the samples were filtered and washed with TCA 10% and ethanol using glass fiber filters. Radioactivity retained in the filters was measured by liquid scintillation counting. For inhibition assays, reaction mixtures were incubated on ice with the scFv C5 antibody. For inhibition reversion, the scFv C5 was preincubated with the H13/R13 peptides for 20 min. After that, reactions were initiated by incubation at 30°C.

### Trypanosome growth and transfection

Bloodstream form of *Trypanosoma brucei* was cultured in HMI-9 medium [24] supplemented with 10% (v/v) fetal calf serum (Sigma-Aldrich), and transfected as described previously [25]. Transfected cells were then cloned by serial dilution in a 24 well microtiter plate. For induction, cells were cultured in the medium above containing 0,1 mg/ml tetracycline and monitored for phenotypic changes (96 hours) or expression levels (72 hours) post induction.

### Data mining and phylogenetic analysis

Ribosomal P protein sequences of *Trypanosoma cruzi, Saccharomyces cerevisiae* and *Homo sapiens* were used to perform BLAST searches [26] against NCBI-GenBank and GeneDB databases, using the tblastn and blastp algorithms. All protein sequences used for the phylogenetic analysis are listed in Table S1. Sequences were aligned with MEGA 4.0 software [27] using the Clustal W algorithm [28] with the PAM protein weight matrix, and all parameters with default settings. A phylogenetic tree was modeled using the minimum evolution method with the pairwise deletion option, and all parameters with default settings. Support for the branching order was determined by 1,000 bootstrap replicates.

### Statistical analysis

Translation inhibition studies were analyzed by two-way ANOVA with Bonferroni's post test. Densitometry was analyzed by one-way ANOVA with Tukey's Multiple Comparison Test. *T. brucei* growth rate was analyzed by two-way ANOVA with Bonferroni's post test. All analysis were done using GraphPad Prism version 5.00 for Windows, GraphPad Software, San Diego California USA, www.graphpad.com.

## Results

### Epitope Specificity

As it was previously reported the scFv C5 (that derives from the monoclonal antibody 17.2) recognizes the C-terminal region of *T. cruzi* ribosomal P2β protein (TcP2β, R13 epitope), and is able to recognize all five *T. cruzi* ribosomal P proteins [16], [29]. Four of them (TcP1α, TcP1β, TcP2α and TcP2β) contain the R13 epitope (**Figure 1A**); and the fifth, TcP0, contains a homologous C-terminal region called P015 (**Figure 1A**). Alanine replacement of R13 peptide showed as important residues for antibody interaction the motif 3-EdDDmGF-9 [29] (**Figure 1A** **, white letters**). The mammalian counterpart epitope (H13) possesses one single, non-conservative amino acid change in the first key glutamic acid position (**Figure 1A**). Therefore, using surface plasmon resonance (SPR) analysis, we tested the ability of the three peptides to inhibit the interaction between the scFv C5 and TcP2β. Inhibition curves were performed using recombinant TcP2β protein fixed to a sensor chip and the scFv C5 in solution together with increasing concentrations of R13, P015 and H13 peptides (**Figure 1B**). Complete inhibition curves could be only obtained with R13 (IC_50_ = 2,37±0.23 µM) (Rsqr = 0.992) and P015 (IC_50_ = 22,64±2.03 µM) (Rsqr = 0.997) peptides but not with the H13 peptide. These results demonstrated the low affinity of scFv C5 for the mammal epitope. Interestingly, both P015 and H13 peptides possess one amino acid change in this motif but the replacement of glycine 8 by aspartic acid in P015 peptide seemed to be less essential than the replacement of glutamic 3 by serine in H13 peptide for scFv C5 interaction.

The crystallographic structure of mAb 17.2 in complex with R13 peptide has been recently solved (PDB 3SGE) [17]. *In silico* replacement of GLU3 by SER showed a strong reduction of the interaction energies that fall from −326.6 kcal/mol (R13 complex) to −204.9 kcal/mol (H13 complex) (**Figure 1C and D**). Particularly, there were three hydrogen bonds established between GLU3 and CDR-H2 residues (ARG-52, SER-53 and ASN-56) that were completely lost by the replacement by SER. In addition H13 peptide, but not R13, was able to acquire a stable conformation in solution using NMR [30], indicating that a strong conformational change is needed for the transition from the free solution form of H13 to the antibody bound conformation (**Figure 1E**) been energetically more expensive than binding to R13 peptide.

### Translation inhibition

**Figure 2:**
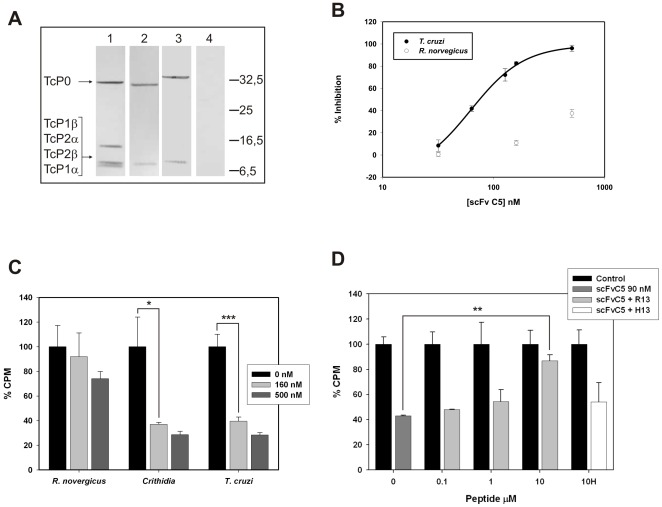
Translation inhibition. **A.** Crude extracts from *T. cruzi* (lane 1), *T. brucei* (lane 2), *C. fasciculata* (lane 3) and *R. norvegicus* (lane 4) were analyzed by SDS-PAGE and Western blot with scFv C5. The position of previously characterized P proteins from *T. cruzi* (Tc) is shown on the left. The image is representative of three independent assays. **B.** Dose-response effect of scFv C5 on protein synthesis by ribosome extracts from *R. norvegicus* and *T. cruzi*. **C.** Effect of scFv C5 160 nM on *in vitro* protein synthesis in ribosome extracts from *R. norvegicus*, *C. fasciculata* and *T. cruzi* compared with the translation inhibitor emetine at 0.1 mg/mg. Average values for control assays were 80,000 cpm, 48,000 cpm and 43,000 cpm for *R. norvegicus*, *C. fasciculata* and *T. cruzi*, respectively. The scFv C5 significantly inhibited protein synthesis by *C. fasciculata* and *T. cruzi* ribosomes (*, p<0.05; ***, p<0.001). **D.** Effect of the preincubation with R13 or H13 peptide on the translation inhibition by scFv C5 (**; p<0.01).

We analyzed the ability of scFv C5 to detect the ribosomal P proteins of *T. cruzi*, *T. brucei*, *C. fasciculata* and *R. norvegicus* by western blot. As it was previously shown [16] the scFv C5 strongly reacted with the *T. cruzi* P0, P1 and P2 proteins (**Figure 2A** **, lane 1**). The sequence of the C-terminal peptide of *T. brucei* shows a conservative change of Glu3 by Asp in three of the low molecular weight P proteins (**Table S2**), and the sequences of *C. fasciculata* P proteins are not known. **Figure 2A** shows that **scFv C5** clearly detected the P proteins on *T. brucei* and *C. fasciculata* extracts (**Figure 2A** **, lanes 2 and 3 respectively**). Similar results were obtained analyzing equal amounts of purified ribosomes from these different organisms (not shown). In contrast, no protein bands were detected using rat extracts (**Figure 2A** **lane 4**), as was expected from SPR analysis.

Because scFv C5 reacts with the functional domain of the ribosomal P proteins, we used an *in vitro* cell-free translation system to determine the ability of this antibody to inhibit protein synthesis. Background values of radioactivity incorporation in the absence of protein synthesis were obtained in the presence of emetine (a well-characterized elongation inhibitor). It should be mentioned that the only difference among the translation mixtures was the ribosome source, ruling out a possible non-specific effect of scFv C5 on processes upstream from translation like ATP regeneration or amino acid activation. Our ribosome preparations lack initiation activity, as we have verified using the recently characterized IF4A inhibitor hippuristanol [23]. The ribosome concentration was estimated around 130 nM (∼10 units of absorbance at 260 nm per ml). Under these conditions, inhibition of protein synthesis on *T. cruzi* ribosomes by scFv C5 was dose-dependent with an IC_50_ of around 62,4 nM (**Figure 2B**). When rat ribosomes were used, it was not possible to obtain a complete translation inhibition curve even with 520 nM scFv C5 (**Figure 2B**). Moreover, scFv C5 up to 500 nM strongly inhibited the incorporation of [^35^S]-methionine on *T. cruzi* and *C. fasciculata* protein synthesis but had no effect on radioactivity incorporation when rat ribosomes were used (**Figure 2C**). Under the same conditions, it was possible to displace the scFv C5 translation inhibition activity by addition of R13 peptide with a maximal effect at 10 µM. However, peptide H13 did not affect scFv C5 inhibition activity at the same concentration (**Figure 2D**). These observations demonstrate that scFv C5 effectively inhibits the elongation step of protein synthesis as a consequence of its interaction with the C-terminal region of parasite ribosomal P proteins. Moreover, differences in epitope affinity correlate with the selective functional effect observed.

### Intrabody expression in *T. brucei*

**Figure 3:**
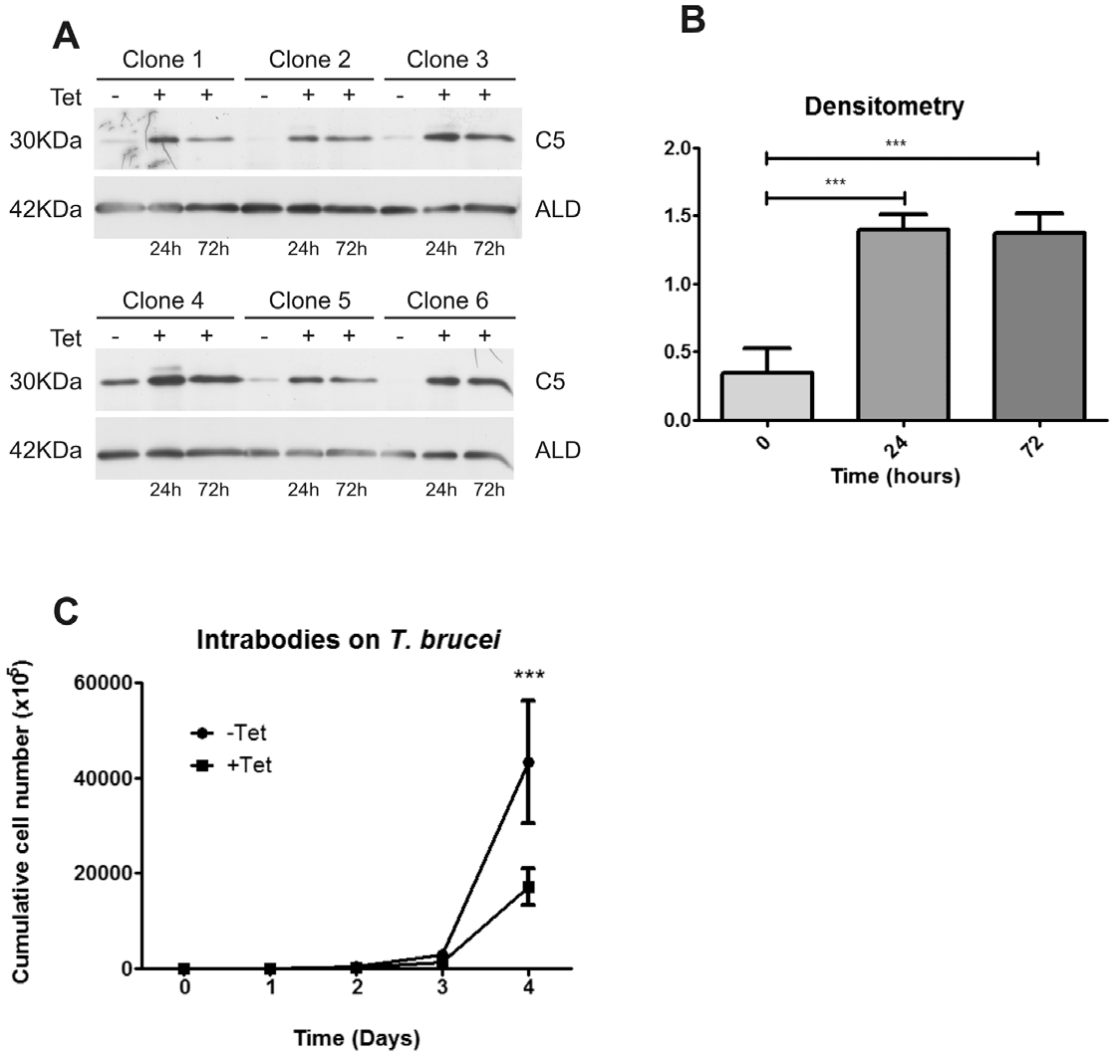
scFv C5 intrabody expression in T. *brucei*. **A.** Western blot detection of C5 intrabody expression at 0, 24 and 72 hours in six different clones. C5 was detected by an anti-Myc antibody. The loading control ALD was detected by an anti-aldolase antibody. **B.** Averaged densitometry analysis of intrabody expression (***; p<0.001). **C.** Averaged growth rate curve of the six different clones induced and un-induced C5 intrabody parasites (***; p<0.001).

At present, there are no confident inducible expression systems available in *T. cruzi* and intrabody transfection using classical *T. cruzi* constitutive expression vectors (pTREX and pRIBOTEX) give no viable clones. Taking advantage from a tightly regulated, inducible expression system in *T. brucei*, we assessed intrabody expression in this organism. We subcloned the scFvC5 coding sequence into the pHD 1700 c-myc vector and transfected it into the *T. brucei* cell line 1313-514. Stable clones were obtained by serial dilution. Tetracycline induction of six different clones gives detectable protein at 24 hours post-induction and almost no detectable protein in the absence of inducer (**Figure 3A**). Intrabody expression levels remained stable until 72 h as can be seen in western blot analysis and the average densitometry analysis (**Figure 3B**). All clones were then subjected to a four day tetracycline induction period comparing the growth rate of induced and uninduced parasites. The averaged growth rate of the six different clones is shown in **figure 3C**. There was a detectable delay in the growth rate starting at day 2 that became statistically different (*p<0.001*) at day 4, indicating a detrimental effect of the intrabody expression on the logarithmic growing phase as can be seen in **figure 3C**. Out of the six clones assessed, clones 2 and 6 that showed no intrabody background expression without tetracycline, were analyzed three times showing reproducible growth rate inhibition after tetracycline induction (**Figure S1**). In addition, luciferase expression or a control intrabody did not affect the growing rate of induced parasites (**Figure S2**).

### Stalk organization and epitope analysis

**Figure 4:**
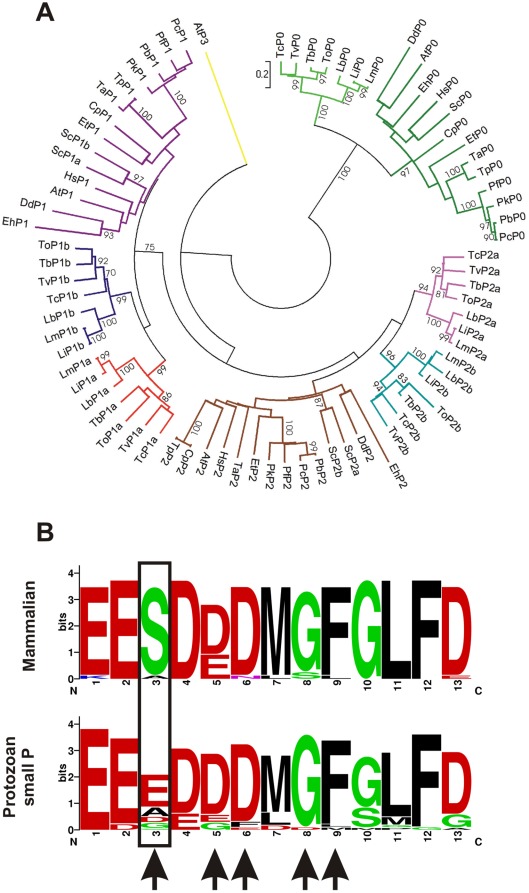
P proteins phylogeny and epitope analysis. **A.** Phylogeny inference of ribosomal P proteins. Red, branch of trypanosomatid P1α proteins. Blue, branch of trypanosomatid P1β proteins. Magenta, branch of trypanosomatid P2α proteins. Light blue, branch of trypanosomatid P2β proteins. Brown, branch of non-trypanosomatid P2 proteins. Violet, branch of non-trypanosomatid P1 proteins. Yellow, *Arabidopsis thaliana* P3. Green, branch of P0 where has been grouped separated the trypanosomatids P0 (light green). Bootstrap values above 70 are shown. **B.** The sequences of the C-terminal residues of mammalian P-proteins and the C-terminal region of the small protozoan P-proteins were aligned using WebLogo [31]. Arrows indicate critical residues for scFv C5 recognition. The region boxed corresponds to the key difference between mammal and protozoan epitopes.

To better understand the organization of the stalk components in different protozoan we generated a sequence alignment (using MEGA 4.0) of the ribosomal P proteins of all genome sequenced protozoan. We searched by homology all ribosomal P proteins (putative and previously reported ones) from the following organisms: *Trypanosoma cruzi* (Tc), *Trypanosoma brucei* (Tb), *Trypanosoma vivax* (Tv), *Trypanosoma congolense* (To), *Leishmania infantum* (Li), *Leishmania braziliensis* (Lb), *Leishmania major* (Lm), *Plasmodium knowlesi* (Pk), *Plasmodium falciparum* (Pf), *Plasmodium berghei* (Pb), *Plasmodium chabaudi* (Pc), *Dictyostelium discoideum* (Dd), *Eimeria tenella* (Et), *Theileria annulata* (Ta), *Theileria parva* (Tp), *Entamoeba histolytica* (Eh) and *Cryptosporidium parvum* (Cp). We also included the ribosomal P proteins of *Homo sapiens* (Hs), *Arabidopsis thaliana* (At) and *Saccharomyces cerevisiae* (Sc) as key markers (**Table S1**). It is important to note that two independent gene duplication events yielded P1 and P2 subtypes (α and β) in trypanosomatids and *S. cerevisiae*. The clusters belonging to trypanosomatids P proteins P0, P1α, P1β, P2α and P2β are all supported by high bootstrap values (Figure 4A). Therefore it is possible that trypanosomatids possess common stalk architecture, probably different from the other analyzed organisms.

Thereafter we generated a sequence alignment of the C-terminal epitope corresponding to different protozoan and mammal organisms (**Table S2**) using weblogo [31]. This allowed a graphical representation of an amino acid multiple sequence alignment showing that in mammals the main amino acid at position 3 is a serine in contrast to protozoan where this position is occupied mainly by glutamic acid, followed by alanine, aspartic acid and finaly glycine (**Figure 4B**). Noteworthy, alanine scanning replacement of R13 peptide showed the importance of glutamic acid 3 for recognition of the antibody [29]. Since the C-terminal region of *Leishmania* P proteins posses an alanine in position 3, it rules out the possibility of an effect of the C5 antibody on this organism.

## Discussion

In the present work, we demonstrated a strong correlation between the affinity of scFv C5 for the C-terminal end of ribosomal P proteins from different species and its ability to inhibit translation in a cell-free system. The difference on IC_50_ observed by SPR (R13 vs H13) together with *in silico* structural analysis based on the recently solved crystallographic structure of the complex Fab 17.2-R13 peptide; clarify how the scFv C5 selectively blocks protein synthesis by trypanosomatid ribosomes. *Western blot* and sequence data analysis showed that different trypanosomatids posses the same C-terminal epitope, all being potentially susceptible to scFv C5 activity. When *T.cruzi* cells were transfected for constitutive expression of scFv C5 intrabody it was not possible to complete the selection period because all cells died. Profiting from the availability of inducible vectors for *T. brucei* and that scFvC5 recognizes *T. brucei* P proteins in *Western Blot*, we tested the effect of scFv C5 intrabody induction on these cells. The scFv C5 intrabody could be detected at protein level using its myc tag at 24 hs post-induction. Induced parasites showed a delay in growth rate, consistent with a blockade of ribosomal translation activity. As can be seen on **figure S1**, it is difficult to establish a relationship between intrabody expression level and functional effect. The inhibition mechanism can be resumed as a protein (scFvC5) that needs to be translated before binding the ribosomal P proteins and thus inhibiting protein synthesis. It means that it is really difficult to obtain total inhibition because the synthesis of the intrabody will also be blocked. We think that the most probable scenario could be interpreted as an equilibrium between scFv translation, protein activity and growth inhibition. In addition, the scFv C5 was able to inhibit *in vitro* protein synthesis in ribosome extracts from *T. brucei* at a similar extent than *T. cruzi* ribosome extracts (**Figure S3**). Intrabodies have been successfully expressed in different cell types [32], [33], [34], [35]. To our knowledge, in this work we have described for the first time the expression of an intrabody in trypanosomatid cells. This knock-down strategy has some potential advantages over RNAi for studying genes that have very high mRNA-turnover rates or whose protein products have very low turnover rates, because they directly interact with the target protein, being less affected by target dynamics. Because intrabodies act at the protein level, they will be of value to specifically block the function of protein complexes and to model drug–target interactions [36].

Sequence analysis showed that mammalian ribosomal P proteins possess a conserved C-terminal region slightly different from the protozoan one, being the main difference the presence of a serine in position 3 (**Figure 4**). This residue showed to be a key residue in scFv C5 recognition, as has been shown all along this work. If this is so, this antibody can potentially inhibit translation activity in different parasitic protozoan cells without affecting the host cell. Moreover, these results reveal the functional C-terminal end of trypanosomatid ribosomal P proteins as a possible specific target for chemotherapy. In addition, the paratope-forming residues from mAb 17.2 recently crystallized (PDB 3SGE) [17] could be an interesting starting point for designing peptide mimetics [37], [38], [39], [40] as specific inhibitors of trypanosomatid translation.

## Supporting Information

Figure S1 **Growth curves of all clones assessed for intrabody expression.** The growth curves and expression profile of each individual clone are shown in every row. On the left side we present the western blots of scFvC5 expression performed at 0 and 24 hours post-induction. The averaged growth curve and densitometry is shown at the bottom and corresponds to the results showed in figure 3. The growth curves of three replicates of clones 2 and 6 and the average growth curve are also shown. Red squares correspond to averaged growth curves. (***; p<0.001).
(TIF)

Figure S2 **Control intrabody expression in T.** ***brucei*** **.** Growth rate of *T. brucei* transfected with the control scFv S3, scFv C5 or Luciferase in presence of tetracycline using the pLew inducible system.
(TIF)

Figure S3 **Translation inhibition.** Effect of scFv C5 50 nM on in vitro protein synthesis in ribosome extracts from *T. cruzi* and *T. brucei* compared with the translation inhibitor emetine at 0.1 mg/mg. Average values for control assays were 6,000 cpm and 19,000 cpm for *T. cruzi* and *T. brucei*, respectively.
(TIF)

Table S1 **Ribosomal P-proteins analyzed in** **Figure 4A** **.**
(DOC)

Table S2 **C-terminal region of the ribosomal P-proteins analyzed in** **Figure 4B** **.** A. Different mammalian P-proteins C-terminal sequences potentially hosts of parasitic infections. B. Protozoan P1/P2 C-terminal sequences.
(DOC)
