# Supplementary figures and images for: Selective Blockade of Trypanosomatid Protein Synthesis by a Recombinant Antibody Anti-Trypanosoma cruzi P2β Protein
Source: PLoS One. 2012 May 3;7(5):e36233. doi: 10.1371/journal.pone.0036233 (PMC3343115; doi:10.1371/journal.pone.0036233)

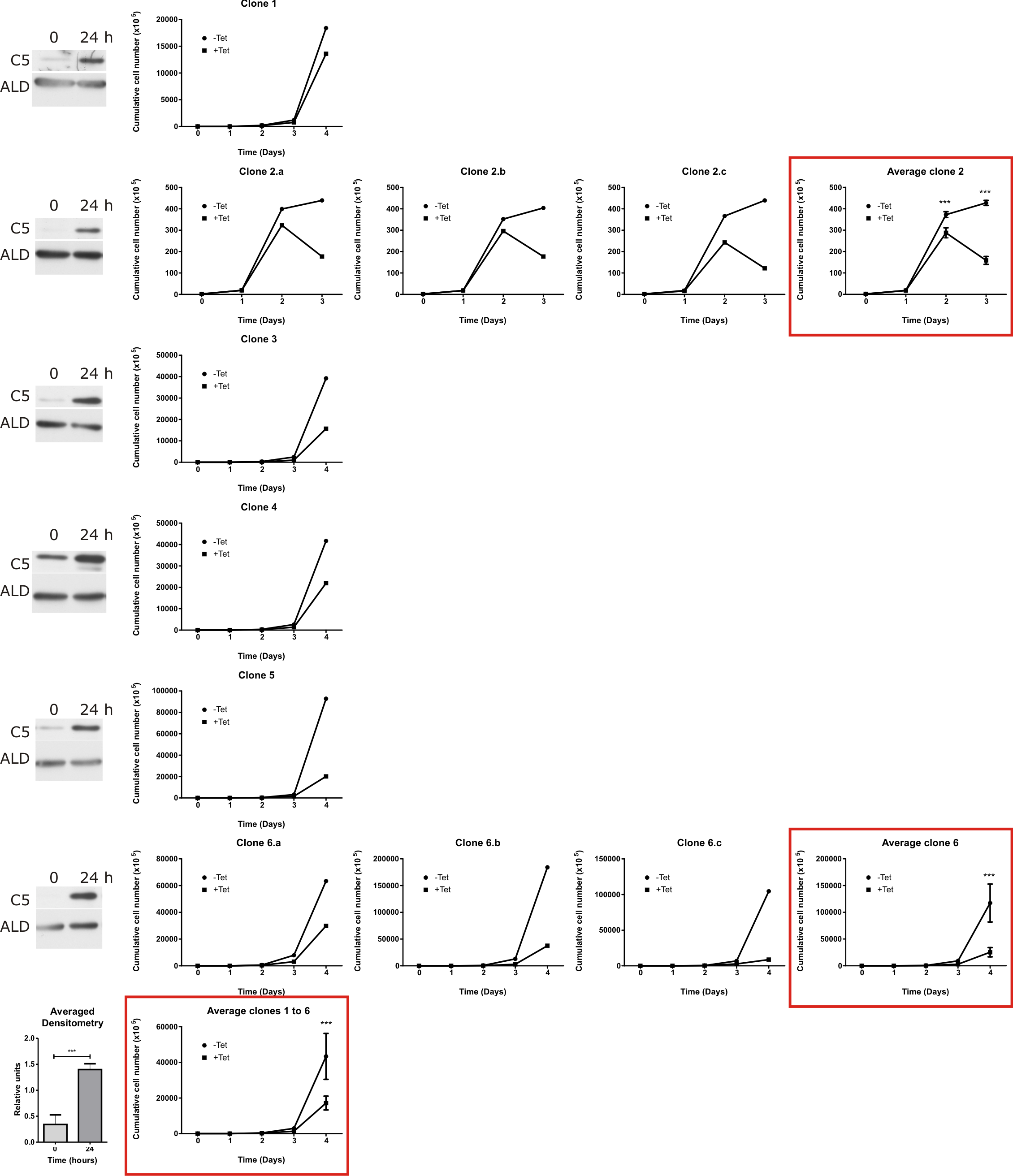

Supplement: Figure S1 — Growth curves of all clones assessed for intrabody expression. The growth curves and expression profile of each individual clone are shown in every row. On the left side we present the western blots of scFvC5 expression performed at 0 and 24 hours post-induction. The averaged growth curve and densitometry is shown at the bottom and corresponds to the results showed in figure 3. The growth curves of three replicates of clones 2 and 6 and the average growth curve are also shown. Red squares correspond to averaged growth curves. (***; p<0.001). (TIF) [file pone.0036233.s001.tif]

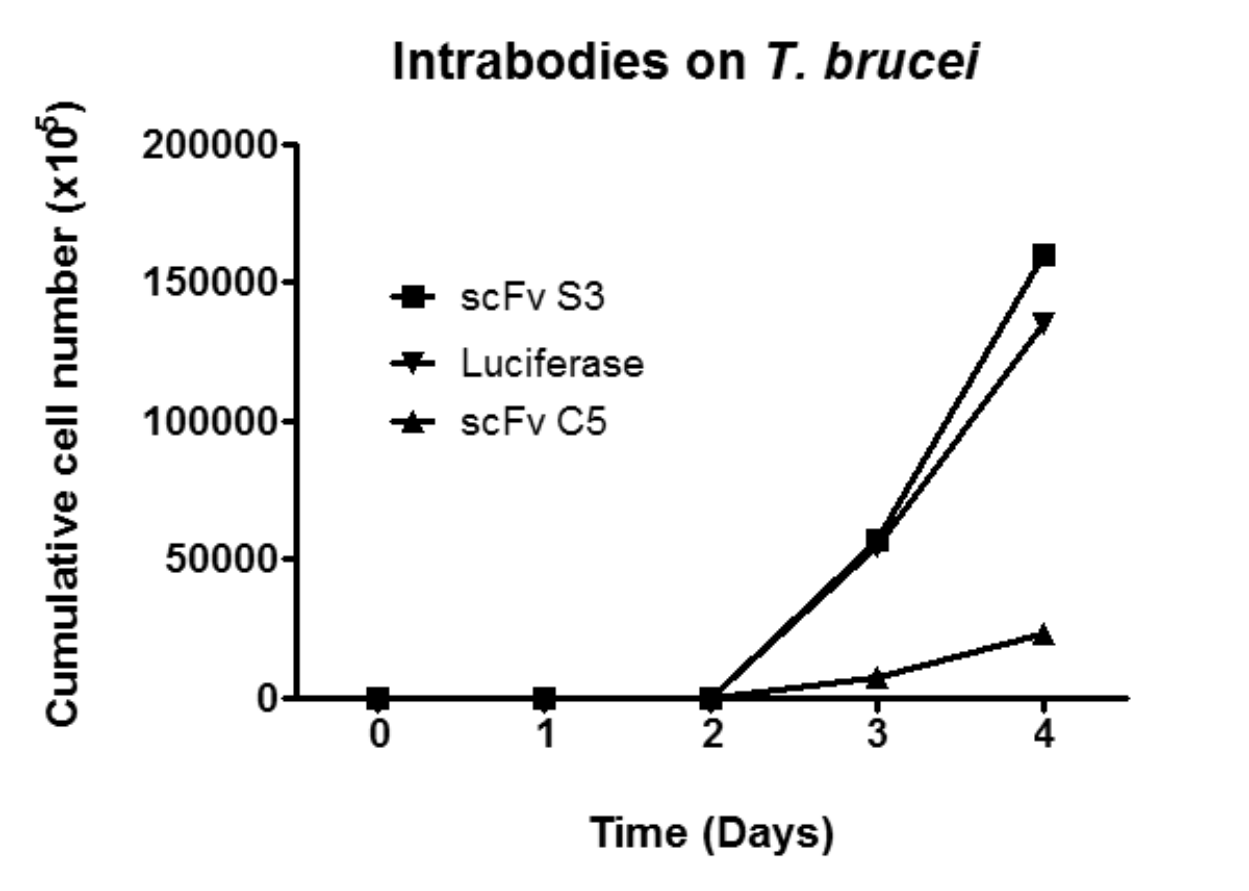

Supplement: Figure S2 — Control intrabody expression in T. brucei . Growth rate of T. brucei transfected with the control scFv S3, scFv C5 or Luciferase in presence of tetracycline using the pLew inducible system. (TIF) [file pone.0036233.s002.tif]

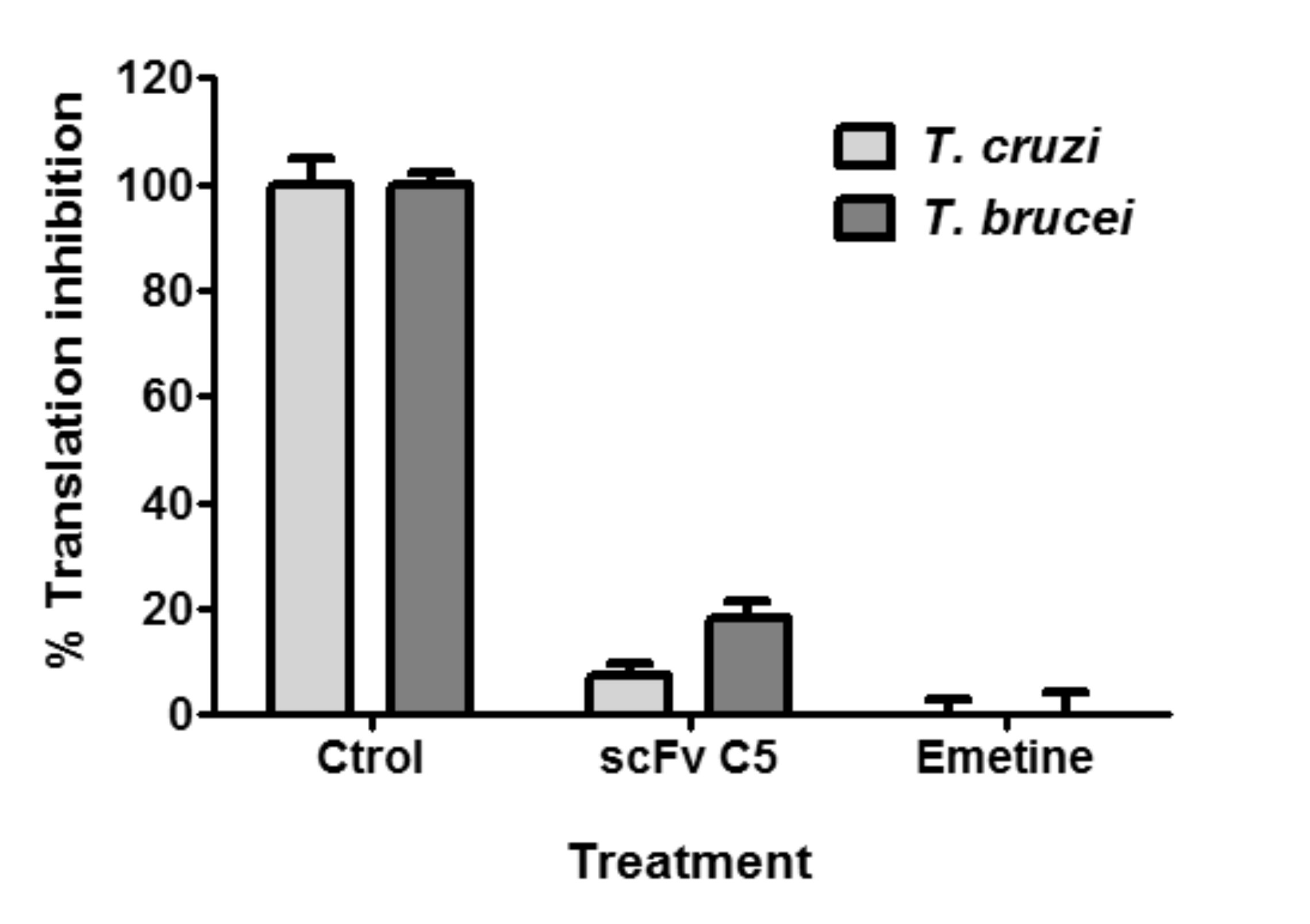

Supplement: Figure S3 — Translation inhibition. Effect of scFv C5 50 nM on in vitro protein synthesis in ribosome extracts from T. cruzi and T. brucei compared with the translation inhibitor emetine at 0.1 mg/mg. Average values for control assays were 6,000 cpm and 19,000 cpm for T. cruzi and T. brucei, respectively. (TIF) [file pone.0036233.s003.tif]
