# Supplementary material for: Selective Blockade of Trypanosomatid Protein Synthesis by a Recombinant Antibody Anti-Trypanosoma cruzi P2β Protein
Source: PLoS One. 2012 May 3;7(5):e36233. doi: 10.1371/journal.pone.0036233 (PMC3343115; doi:10.1371/journal.pone.0036233)
Supplement: Table S1 — Ribosomal P-proteins analyzed in Figure 4A . (DOC) [file pone.0036233.s004.doc]

| Organism | Abbreviation | Accesion Number | Original Identification | Data base |
| --- | --- | --- | --- | --- |
| *Trypanosoma cruzi* | TcP0 | X65066 | Acidic ribosomal P0 protein | Blast |
|  | TcP1α | X65025 | ribosomal P1a type protein | Blast |
|  | TcP1β | AY618551 | ribosomal P protein type 1 | Blast |
|  | TcP2α | X65065 | ribosomal P2-type protein | Blast |
|  | TcP2β | X52323 | ribosomal P protein | Blast |
| *Trypanosoma brucei* | TbP0 | XM_823196 | 60S acidic ribosomal subunit protein | Blast |
|  | TbP1α | XM_839763 | 60S acidic ribosomal protein | Blast |
|  | TbP1β | XM_817495 | 60S acidic ribosomal protein P2 | Blast |
|  | TbP2α | XM_821871 | acidic ribosomal protein | Blast |
|  | TbP2β | XM_840536 | acidic ribosomal protein P2 | Blast |
| *Trypanosoma congolense* | ToP0 | AB056702 | ribosomal P0 subunit protein | Blast |
|  | ToP1α | congo1540a08.q1k_0 | 60S acidic ribosomal protein, putative | GeneDB |
|  | ToP1β | congo1321a09.p1k_2 | 60S acidic ribosomal protein, putative | GeneDB |
|  | ToP2α | congo842b09.p1k_0 | 60S acidic ribosomal protein P2, putative | GeneDB |
|  | ToP2β | congo364e01.q1k_22 | 60S acidic ribosomal protein P2, putative | GeneDB |
| *Trypanosoma vivax* | TvP0 | tviv891a06.q1k_2 | 60S acidic ribosomal subunit protein, putative | GeneDB |
|  | TvP1α | tviv1260a09.p1k_0 | 60S acidic ribosomal protein, putative | GeneDB |
|  | TvP1β | tviv529g08.q1k_8 | 60S acidic ribosomal protein P2, putative | GeneDB |
|  | TvP2α | tviv1010e05.q1k_3 | 60S acidic ribosomal protein, putative | GeneDB |
|  | TvP2β | tviv101d07.q1k_5 | 60S acidic ribosomal protein P2, putative | GeneDB |
| *Leishmania major* | LmP0 | XM_843003 | 60S acidic ribosomal subunit protein | Blast |
|  | LmP1α | XM_001681903 | 60S acidic ribosomal protein, putative | Blast |
|  | LmP1β | XM_001687285 | 60S acidic ribosomal protein P2, putative | Blast |
|  | LmP2α | XM_001681983 | 60S acidic ribosomal protein P2 | Blast |
|  | LmP2β | XM_001684931 | 60S acidic ribosomal protein P2, putative | Blast |
| *Leishmania braziliensis* | LbP0 | XM_001565859 | 60S acidic ribosomal subunit protein, putative | Blast |
|  | LbP1α | CBZ14476 | putative 60S acidic ribosomal protein | Blast |
|  | LbP1β | XM_001561580 | 60S acidic ribosomal protein P2, putative | Blast |
|  | LbP2α | XM_001563513 | 60S acidic ribosomal protein P2 | Blast |
|  | LbP2β | XM_001566949 | 60S acidic ribosomal protein P2, putative | Blast |
| *Leishmania infantum* | LiP0 | XM_001466337 | 60S acidic ribosomal subunit protein | Blast |
|  | LiP1α | XM_001464343 | 60S acidic ribosomal protein, putative | Blast |
|  | LiP1β | XM_001462759 | 60S acidic ribosomal protein P2, putative | Blast |
|  | LiP2α | X68016 | acidic ribosomal protein LiP | Blast |
|  | LiP2β | XM_001467131 | 60S acidic ribosomal protein P2 | Blast |
| *Plasmodium falciparum* | PfP0 | XM_001347948 | ribosomal phosphoprotein P0 | Blast |
|  | PfP1 | XM_001347683 | 60S acidic ribosomal protein P1 | Blast |
|  | PfP2 | XM_001351133 | 60S acidic ribosomal protein P2 | Blast |
| *Plasmodium knowlesi* | PkP0 | XM_002259271 | ribosomal phosphoprotein | Blast |
|  | PkP1 | XM_002258995 | 60s acidic ribosomal protein p1 | Blast |
|  | PkP2 | XM_002258915 | 60S Acidic ribosomal protein P2 | Blast |
| *Plasmodium berghei* | PbP0 | AY099370 | ribosomal phosphoprotein P0 | Blast |
|  | PbP1 | XM_670358 | 60S acidic ribosomal protein p1 | Blast |
|  | PbP2 | XM_672083 | 60S Acidic ribosomal protein P2 | Blast |
| *Plasmodium chabaudi* | PcP0 | XM_736891 | ribosomal phosphoprotein P0 | Blast |
|  | PcP1 | Contig chab09 |  | GeneDB |
|  | PcP2 | XM_736917 | 60S Acidic ribosomal protein P2 | Blast |
| *Theilera parva* | TpP0 | XM_760728 | 60S acidic ribosomal protein, P0 | Blast |
|  | TpP1 | XM_759254 | 60S acidic ribosomal protein P1 | Blast |
|  | TpP2 | XM_759843 | 60S acidic ribosomal protein P2 | Blast |
| *Theilera annulata* | TaP0 | XM_949337 | ribosomal phosphoprotein P0 | Blast |
|  | TaP1 | XM_948255 | 60S ribosomal protein P1, putative | Blast |
|  | TaP2 | XM_947253 | 60S acidic ribosomal protein p2 | Blast |
| *Cryptosporidium parvum* | CpP0 | XM_625816 | ribosomal protein P0 like protein of the L10 family | Blast |
|  | CpP1 | XM_626174 | 60S acidic ribosomal protein LP1 like protein | Blast |
|  | CpP2 | XP_625382 | 60S acidic ribosomal protein LP2 | Blast |
| *Eimeria tenella* | EtP0 | AF353516 | ribosomal protein P0 | Blast |
|  | EtP1 | TWINSCAN_  PHASES00000242140 | ribosomal protein P1, putative | GeneDB |
|  | EtP2 | AF353514 | ribosomal protein P2 | Blast |
| *Entamoeba histolytica* | EhP0 | XM_652489 | 60S acidic ribosomal protein P0,putative | Blast |
|  | EhP1 | XM_647546 | 60S acidic ribosomal protein P1, putative | Blast |
|  | EhP2 | XM_650477 | 60S acidic ribosomal protein P2,putative | Blast |
| *Dictyostelium discoideum* | DdP0 | X56194 | ribosomal acidic phosphoprotein P0 | Blast |
|  | DdP1 | X56193 | ribosomal acidic phosphoprotein P1 | Blast |
|  | DdP2 | X56192 | ribosomal acidic phosphoprotein P2 | Blast |
| *Arabidopsis thaliana* | AtP0 | NM_129559 | 60S acidic ribosomal protein P0 | Blast |
|  | AtP1 | NM_099992 | 60S acidic ribosomal protein P1 | Blast |
|  | AtP2 | NM_128331 | 60S acidic ribosomal protein P2 | Blast |
|  | AtP3 | NM_118722 | 60S acidic ribosomal protein P3 | Blast |
| *Homo sapiens* | HsP0 | M17885 | acidic ribosomal phosphoprotein P0 | Blast |
|  | HsP1 | M17886 | acidic ribosomal phosphoprotein P1 | Blast |
|  | HsP2 | M17887 | acidic ribosomal phosphoprotein P2 | Blast |
| *Saccharomyces cerevisiae* | ScP0 | M26506 | L10e protein gene | Blast |
|  | ScP1α | M26504 | L12eIIA protein gene | Blast |
|  | ScP1β | M26507 | L12eIIB protein gene | Blast |
|  | ScP2α | M26503 | L12eIB protein gene | Blast |
|  | ScP2β | M26505 | L12eIA protein gene | Blast |
