# Supplementary material for: Selective Blockade of Trypanosomatid Protein Synthesis by a Recombinant Antibody Anti-Trypanosoma cruzi P2β Protein
Source: PLoS One. 2012 May 3;7(5):e36233. doi: 10.1371/journal.pone.0036233 (PMC3343115; doi:10.1371/journal.pone.0036233)
Supplement: Table S2 — C-terminal region of the ribosomal P-proteins analyzed in Figure 4B . A. Different mammalian P-proteins C-terminal sequences potentially hosts of parasitic infections. B. Protozoan P1/P2 C-terminal sequences. (DOC) [file pone.0036233.s005.doc]

A. Different mammalian P-proteins C-terminal sequences potentially hosts of parasitic infections.

| Rattus norvegicus  Homo sapiens  Mus musculus  [*Bos taurus*](http://www.ncbi.nlm.nih.gov/mapview/map_search.cgi?taxid=9913)  [*Canis familiaris*](http://www.ncbi.nlm.nih.gov/mapview/map_search.cgi?taxid=9615)  [*Felis catus*](http://www.ncbi.nlm.nih.gov/mapview/map_search.cgi?taxid=9685)  [*Macaca mulatta*](http://www.ncbi.nlm.nih.gov/mapview/map_search.cgi?taxid=9544)  [*Monodelphis domestica*](http://www.ncbi.nlm.nih.gov/mapview/map_search.cgi?taxid=13616)  [*Ovis aries*](http://www.ncbi.nlm.nih.gov/mapview/map_search.cgi?taxid=9940)  [*Pan troglodytes*](http://www.ncbi.nlm.nih.gov/mapview/map_search.cgi?taxid=9598)  [*Sus scrofa*](http://www.ncbi.nlm.nih.gov/mapview/map_search.cgi?taxid=9823) | EESDEDMGFGLFD  EESDDDMGFGLFD  EESDEDMGFGLFD  EESDDDMGFGLFD  EESDEDMGFGLFD  EESDDDMGFGLFD  EEADDDMGFGLFD  EESDEDMGFGLFD  EESDDNMSFGLFD  EESDEDMGFGLFD  EESDDDMGFGLFD  EESDEDMGFGLFD  KESDDDMGFGLFD  EESDDDMGFGLFD  EESDDDMGFGLFD  EESDDDLGFGLFD  EESDDDMGFGLFD  EESDEDMGFGLFD  EESDDDMGFGLFD  EESDDDMGFGLFD  EESDEDMGFGLFD  EESDDDMGFGLFE  EESDDDMSFGLFD  EESDDDMGLGLFD |
| --- | --- |

B. Protozoan P1/P2 C-terminal sequences.

|  | **P1** | | **P2** | | **P0** |
| --- | --- | --- | --- | --- | --- |
|  | **alpha** | **beta** | **alpha** | **beta** |
| ***Trypanosoma cruzi*** | EEEDDDMGFGLFD | EEEDDDMGFGLFD | EEEDDDMGFGLFD | EEEDDDMGFGLFD | EDDDDDFGMGALF |
| ***Trypanosoma vivax*** | EEEDDDMGFGLFD | EEEDDDMGFGLFD | EEEDDDMGFGLFD | EEEDDDMGFGLFD | EEEDDDFGMGALF |
| ***trypanosoma congolense*** | EEEDDDMGFGLFD | EEDDDDMGFGLFD | EEEDDDMGFGLFD | EEDDDDMGFGLFD | EEEDDDFGMGALF |
| ***Trypanosoma brucei*** | EEDDDDMGFGLFD | EEDDDDMGFGLFD | EEEDDDMGFGLFD | EEDDDDMGFGLFD | EEDDDDFGMGALF |
| ***Leishmania infantum*** | EDADDDMGFGLFD | EEGDDDMGFGLFD | EEADDDMGFGLFD | EEADDDMGFGLFD | ESDEDDFGMGGLF |
| ***Leishmania major*** | EDADDDMGFGLFD | EEGDDDMGFGLFD | EEADDDMGFGLFD | EEADDDMGFGLFD | ESDEDDFGMGGLF |
| ***Leishmania braziliensis*** | EEADDDMGFGLFD | EEGDDDMGFGLFD | EEADDDMGFGLFD | EEADDDMGFGLFD | ESEEDDFGMGALF |
| ***Eimeria tenella*** | EEEDADMGFSLFD | | EEEDGDMGLSLFD | | EEEDGDMGFSLFD |
| ***Theileria annulata*** | EEEDEDMGFSLFD | | EEEEDDMGFSLFD | | EEEDDDLGFSLFD |
| ***Plasmodium berghei*** | EEEEDDLGFSLFG | | EEEEDDLGFSLFG | | EEEDGFMGFGMFD |
| ***Plasmodium chabaudi*** | EEEEDDLGFSLFG | | EEEEDDLGFSLFG | | EEEDGFMGFGMFD |
| ***Plasmodium knowlesi*** | EEEEDDLGFSLFG | | EEEEDDLGFSLFG | | EEEDGFMGFGMFD |
| ***Plasmodium falciparum*** | EEEEDDLGFSLFG | | EEEEDDLGFSLFG | | EEEDGFMGFGMFD |
| ***Entamoeba histolytica*** | EAEEDFGGFGDLF | | EEEEDFGGFGDLF | | EEEEDFGGFGDLF |
| ***Dictyostelium discoideum*** | EESDDDMDKHVQD | | EESDDDMGMGLFD | | EESDDDMGMGLFD |
| ***Cryptosporidium parvum*** | EEEEGDLGFSLFD | | EEEEGDLGFSLFD | | EEEEGDLGFSLFD |
| ***Theileria parva*** | EEEEGDLGFSLFD | | EEEDEDMGFSLFD | | EEEDEDMGFSLFD |
